# Practical Use of Regularization in Individualizing a Mathematical Model of Cardiovascular Hemodynamics Using Scarce Data

**DOI:** 10.3389/fphys.2020.00452

**Published:** 2020-05-26

**Authors:** Ali Tivay, Xin Jin, Alex Kai-Yuan Lo, Christopher G. Scully, Jin-Oh Hahn

**Affiliations:** ^1^Department of Mechanical Engineering, University of Maryland, College Park, College Park, MD, United States; ^2^Office of Science and Engineering Laboratories, Center for Devices and Radiological Health, U.S. Food and Drug Administration, Silver Spring, MD, United States

**Keywords:** individualization, physiological model, regularization, practical identifiability, volume kinetics, cardiovascular hemodynamics, hemorrhage, resuscitation

## Abstract

Individualizing physiological models to a patient can enable patient-specific monitoring and treatment in critical care environments. However, this task often presents a unique “practical identifiability” challenge due to the conflict between model complexity and data scarcity. Regularization provides an established framework to cope with this conflict by compensating for data scarcity with prior knowledge. However, regularization has not been widely pursued in individualizing physiological models to facilitate patient-specific critical care. Thus, the goal of this work is to garner potentially generalizable insight into the practical use of regularization in individualizing a complex physiological model using scarce data by investigating its effect in a clinically significant critical care case study of blood volume kinetics and cardiovascular hemodynamics in hemorrhage and circulatory resuscitation. We construct a population-average model as prior knowledge and individualize the physiological model *via* regularization to illustrate that regularization can be effective in individualizing a physiological model to learn salient individual-specific characteristics (resulting in the goodness of fit to individual-specific data) while restricting unnecessary deviations from the population-average model (achieving practical identifiability). We also illustrate that regularization yields parsimonious individualization of only sensitive parameters as well as adequate physiological plausibility and relevance in predicting internal physiological states.

## Introduction

Clinical care automation has been a domain of interest for a few decades by virtue of its potential for error-free and vigilant performance of routine and low-level patient monitoring and treatment tasks (Hemmerling et al., 2010; Salinas et al., 2011; Dussaussoy et al., 2014; Rinehart et al., 2015; Brogi et al., 2017; Hundeshagen et al., 2017; Pasin et al., 2017), yet realizing this potential is contingent upon establishing the safety and the performance characteristics of clinical care automation. Patient physiology models built upon physical principles (hereafter called physiological models) can facilitate the development (Jin-Oh et al., 2012; Bighamian et al., 2014; Jin et al., 2018) and the testing (Kovatchev et al., 2009; Ortiz et al., 2010; Brown et al., 2015) of clinical care automation capabilities. However, individualizing physiological models (which can enable the systematic development and the testing of patient-specific clinical care automation) presents formidable challenge due to the conflict between model complexity and data scarcity. That is, physiological models are complex and involve a large number of unknown patient-specific parameters that frequently exhibit interaction properties, whereas clinical data pertaining to an individual patient are scarce in both quantity and quality. For example, blood pressure (BP) can increase in response to an increase in cardiac output (CO) and an increase in total peripheral resistance (TPR), but routine clinical measurements often provide only BP which does not offer sufficient information to uniquely determine CO- and TPR-related model parameters. In fact, such a conflict appears to be a main obstacle in characterizing a wide range of mechanistic models in physiology as well as in broader fields of biology and physics, which has been called the sloppiness property (Transtrum et al., 2010, 2015; Machta et al., 2013; White et al., 2016) or lack of practical identifiability (Raue et al., 2009; Maiwald et al., 2011) in different contexts. If not properly addressed, such conflict may yield a physiological model suffering from non-unique and physically irrelevant parameter values as well as poor individual-specific internal (i.e., unmeasured) state predictions.

The existing body of work has addressed the practical identifiability challenge mainly in two ways: (i) by modifying the model structure or parameterization toward more simplified or lumped models and (ii) by utilizing some form of regularization (or more generally, prior knowledge) to additionally inform the modeling procedure and effectively reduce the complexity of the model search space. Analyzing and modifying the model structure has particularly strong precedents in physiological modeling, where effective tools such as variation-based sensitivity analysis (Sobol, 2001; Saltelli et al., 2008, 2010) and profile-likelihood (Raue et al., 2009, 2014; Maiwald et al., 2016) methods are used to assess the appropriateness of a model structure and the importance of model parameters with respect to given data. While useful in informing potential modifications to the model structure and parameterization (Machta et al., 2013; Mattingly et al., 2018), many of these tools are formalized on a pre-determined level of data availability, making them less powerful if data availability varies (which is quite common in clinical scenarios). Regularization is an established framework to cope with conflicts between model complexity and data scarcity, by which one can potentially compensate for the scarce data available to individualize a physiological model based on prior knowledge. To date, the notion of prior distribution (Toni et al., 2009; Gelman et al., 2013) and its closely related counterpart in regularization (Benning and Burger, 2018) have been pursued in a wide range of deterministic and stochastic modeling problems to incorporate additional information into the modeling problem and control the complexity of the model search space (Davidian and Giltinan, 2003; Tivay et al., 2019; Zhang et al., 2019).

The goal of this work is to garner potentially generalizable insight into the role of regularization in individualizing a complex physiological model using scarce data by studying its effect in a clinically significant critical care case study of blood volume (BV) kinetics and cardiovascular hemodynamics in hemorrhage and circulatory resuscitation. We construct a population-average physiological model as prior knowledge and individualize the physiological model *via* regularization to elucidate how regularization enables the physiological model to capture salient individual-specific characteristics (leading to the goodness of fit to individual-specific data) while restricting unnecessary deviations from prior knowledge (achieving practical identifiability). We also analyze how regularization affects the plausibility and the relevance of the physiological model under varying levels of scarcity in individual-specific data in terms of its parameter values and internal state predictions.

This paper is organized as follows: section “Cardiovascular Hemodynamics in Hemorrhage and Circulatory Resuscitation” outlines the physiological model and the experimental data used in the case study. Section “Materials and Methods” describes a step-by-step procedure for applying regularization to individualize the physiological model. Section “Results” presents the results, which are discussed in section “Discussion”. Section “Conclusions” concludes the paper with suggested future work.

## Cardiovascular Hemodynamics in Hemorrhage and Circulatory Resuscitation

Credible physiological models of BV kinetics and cardiovascular hemodynamics have the potential to facilitate the development and the testing of circulatory resuscitation devices and algorithms (Parvinian et al., 2018). Prior work has reported a wide spectrum of physiological models (in terms of scale and detail) to represent volume kinetics (Hahn, 2010; Bighamian et al., 2016) and cardiovascular hemodynamics (Moss et al., 2012; Bighamian et al., 2018) in response to stimuli. In some cases, these physiological models were also analyzed for practical identifiability (Moss et al., 2012; Marquis et al., 2018; Pathmanathan et al., 2019; Pironet et al., 2019). In this work, we employ an extended and enhanced version of a physiological model reported in our prior work (Bighamian et al., 2018), which concerns the effect of hemorrhage and circulatory resuscitation on the changes in BV kinetics and cardiovascular hemodynamics.

### Model Description

**FIGURE 1:**
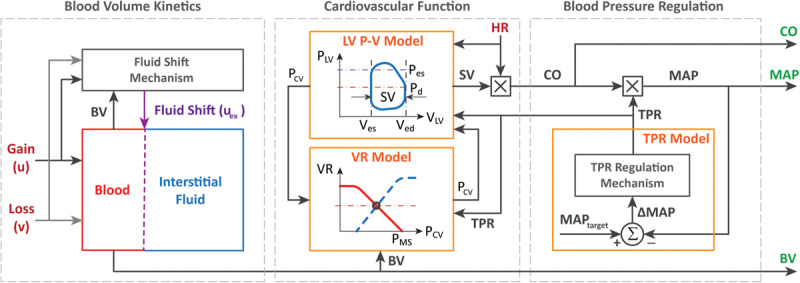
Physiological model of blood volume kinetics and cardiovascular hemodynamics in response to hemorrhage and circulatory resuscitation.

The physiological model is composed of component mathematical models that represent BV kinetics, cardiovascular function, and BP regulation, all at the systems level (Figure 1).

The BV kinetics model is a lumped-parameter model that simulates the effect of fluid gain, blood loss, and inter-compartmental fluid exchange on changes in BV. In this model, the time rate of change in BV (Δ*V*_*B*_) can be described by the following differential equation:

(1) $$\Delta \,{\dot{V}}_{B}\,\left(t\right)=u\,\left(t\right)-v\,\left(t\right)-u_{e\,x}\,\left(t\right)$$

where *u*, *v*, and *u*_*ex*_, respectively, denote the rate of external fluid gain (e.g., fluid resuscitation), external fluid loss (e.g., hemorrhage and urinary output), and internal fluid exchange between BV and interstitial tissue compartments. The rate of fluid exchange is macroscopically modeled as a control input signal that regulates BV to a target value *r*_*BV*_ as follows:

(2) $$u_{e\,x}\,\left(t\right)=-K_{p}\,\left[r_{B\,V}\,\left(t\right)-\text{Δ}\,V_{B}\,\left(t\right)\right]$$

where *K*_*p*_ is the controller gain and *r*_*BV*_ represents the target change in BV, which is computed according to the history of external fluid gain and loss as follows:

(3) $$r_{B\,V}\,\left(t\right)=\frac{1}{1+\alpha_{u}}\,\int_{0}^{t}u\,\left(\tau \right)\,d\,\tau -\frac{1}{1+\alpha_{v}}\,\int_{0}^{t}v\,\left(\tau \right)\,d\,\tau$$

where the ratio $\frac{1}{1+a_{u}}$ determines the fraction of the total external fluid gain that eventually remains within the BV compartment (with the rest shifted to the interstitial tissue compartment), and the ratio $\frac{1}{1+a_{v}}$ determines the fraction of the total fluid loss that is not compensated for by a refill from the interstitial tissue compartment. In our prior work, we demonstrated that the macroscopic expressions above can reproduce BV kinetic responses to volume perturbations with crystalloid, colloid, and blood (Bighamian et al., 2016, 2018).

The cardiovascular function model is built to compute CO from BV based on the CO–venous return equilibrium principle (Guyton et al., 1975) and the left ventricular (LV) pressure–volume relationship (Lankhaar et al., 2009). The details of such a derivation can be found in our previous work (Bighamian et al., 2018), which yields the following model relating BV to CO:

(4) $$CO\left(t\right)=\frac{H\,R\,\left(t\right)}{A+\frac{A}{E_{s}}\,H\,R\,\left(t\right)\,T\,P\,R\,\left(t\right)}\log(-\frac{\gamma \,k}{B}TPR\left(t\right)CO\left(t\right)+\frac{\gamma }{B\,C_{s}}V_{B}\left(t\right)-\frac{\gamma }{B\,C_{s}}\eta V_{B\,0}+1)$$

where *HR*(*t*) is the heart rate, *TPR*(*t*) is the TPR associated with the arterial circulation, *C*_*s*_ is the systemic capacitance, *E*_*s*_ is the left ventricular elastance, *A* and *B* define the shape of the LV pressure–volume loop, γ represents the approximate proportionality constant between central venous (*P*_*CV*_) and LV end diastolic (*P*_*LVED*_) pressures (γ≈*P*_*LVED*_/*P*_*CV*_), *k* denotes the ratio between the resistance to venous return and TPR (*k* = *R*_*VR*_/*TPR*), *V*_*B0*_ is the initial BV, and η represents a proportional relationship between *V*_*B0*_ and the unstressed BV *V*_*BU*_: *V*_*BU*_ = η*V*_*B*0_.

The BP regulation component of the model approximates the regulatory mechanism in the body that monitors the discrepancy between a pre-specified, intrinsic mean arterial BP (MAP) set point (*MAP*_*target*_) *versus* the subject’s actual MAP (*MAP*(*t*)) and adjusts TPR (and accordingly, the resistance to venous return) to maintain MAP near the set point level. This component is modeled with the following equations:

(5a) $$M\,A\,P\,\left(t\right)=C\,O\,\left(t\right)\,T\,P\,R\,\left(t\right)=C\,O\,\left(t\right)\,\left[T\,P\,R_{0}+\text{Δ}\,T\,P\,R\,\left(t\right)\right]$$

(5b) $$\text{Δ}\,\dot{T}\,P\,R\left(t\right)+p_{T\,P\,R}\,\text{Δ}\,T\,P\,R\,\left(t\right)=k_{T\,P\,R}\,\left[M\,A\,P_{\text{target}}-M\,A\,P\,\left(t\right)\right]$$

where *TPR*_*0*_ is the initial value of TPR, which can be found from the initial value of CO and MAP (*TPR*_0_ = *MAP*_0_/*CO*_0_), *k*_*TPR*_ is the gain of MAP regulation, and *p*_*TPR*_ represents the speed of MAP regulation. Due to the relatively short time period (180 min) associated with the dataset as well as the presumably maximal level of short-term BP regulation responses due to large initial hemorrhage, the full mechanistic details of BP regulation mechanisms (e.g., the long-term aspects associated with the responses of the renin–angiotensin–aldosterone and anti-diuretic hormone systems) are not considered, and the collective short-term effects of BP regulation mechanisms are macroscopically expressed by Eq. 5.

### Experimental Dataset

The experimental measurements acquired from animals in an array of prior work (Rafie et al., 2004; Vaid et al., 2006; Marques et al., 2017) were used as data in this case study. A total of *N* = 23 sheep constituted the population considered in this study. The data included the time series sequences of BV, CO, and MAP acquired from these sheep subjects. In each sheep, initial BV was measured using a dye concentration method. Then, subsequent changes in BV were measured with hemoglobin dilution after correction for the loss of red blood cells. CO was measured with a flow probe placed on the pulmonary artery. MAP was measured with arterial catheterization. These signals were measured at 5-min intervals during a measurement period of 180 min. Each sheep was subjected to a large initial hemorrhage (25 ml/kg; see “H” at 0–15 min in Figure 4) followed by resuscitation (see “I” at 30–180 min in Figure 4) and subsequent minor hemorrhages (5 ml/kg; see “H” at 50–55 and 70–75 min in Figure 4). Fluid resuscitation was performed by previously developed closed-loop control algorithms (Rafie et al., 2004; Vaid et al., 2006; Marques et al., 2017) designed to maintain MAP. Hemorrhage and fluid resuscitation yielded diverse BV, CO, and MAP responses in the datasets, which is appropriate to analyze the role of regularization in individualizing the physiological model (Table 1).

**TABLE 1:** Range of blood volume (BV), cardiac output (CO), and mean arterial BP (MAP) responses at important time instants during the experimental protocol [mean (SD), *N* = 23].

**Time (min)**	**BV (L)**	**CO (L/min)**	**MAP (mmHg)**
Baseline pre-hemorrhage (*t=0*)	2.37 (0.44)	4.40 (0.96)	91.22 (10.60)
Immediate post-hemorrhage (*t=15*)	1.83 (0.44)	2.23 (0.83)	52.33 (16.73)
Start of resuscitation (*t=30*)	1.86 (0.41)	2.21 (0.79)	53.17 (10.71)
End of resuscitation (*t=180*)	2.22 (0.58)	4.67 (1.15)	82.80 (6.31)

## Materials and Methods

In this section, we present a procedure for applying regularization to the physiological model outlined in section “Cardiovascular Hemodynamics in Hemorrhage and Circulatory Resuscitation”. Special emphasis is given to the construction of prior knowledge, which involves the choice of a regularization function employed in individualizing the physiological model. Then, data analysis details are presented.

### Individualizing the Physiological Model

Given the data associated with an individual subject *i*, the inputs given to the subject (i.e., hemorrhage and fluid resuscitation) are denoted by $U_{i}^{d}=\left\{u\,\left(t\right),v\,\left(t\right)\right\}$, while the outputs of the subject are denoted by a vector $Y_{i}^{d}$. For instance, in case BV, CO, and MAP data are available in the individual *i*, then $Y_{i}^{d}$ is defined as:

(6) $$Y_{i}^{d}=[BV_{i}^{d}\left(t_{1},\ldots ,t_{n_{B\,V}}\right)CO_{i}^{d}\left(t_{1},\ldots ,t_{n_{C\,O}}\right)MAP_{i}^{d}\left(t_{1},\ldots ,t_{n_{M\,A\,P}}\right)]$$

where $X_{i}^{d}\,\left(t_{1},\ldots ,t_{n_{X}}\right)$ is a row vector containing the measurements of *X* at times (*t*_1_,…,*t*_*n*_*X*__). The nominal noise covariance matrix associated with $Y_{i}^{d}$ is $\text{Σ}=d\,i\,a\,g\,\left(\sigma_{B\,V}^{2}\,I_{n_{B\,V}},\sigma_{C\,O}^{2}\,I_{n_{C\,O}},\sigma_{M\,A\,P}^{2}\,I_{n_{M\,A\,P}}\right)$.

To obtain model predictions $Y_{i}\,\left(\theta ,U_{i}^{d}\right)$ corresponding to the elements of the data vector $Y_{i}^{d}$, the model equations in section “Cardiovascular Hemodynamics in Hemorrhage and Circulatory Resuscitation” are numerically solved given the inputs $U_{i}^{d}$ and the vector of lumped model parameters θ, which is defined as follows (see Appendix Table A1 for details and ranges):

(7) $$\theta =\left[\alpha_{u}\,\alpha_{v}\,K_{p}\,A\,\frac{A}{E_{s}}\,\frac{\gamma \,k}{B}\,\frac{\gamma }{B\,C_{s}}\,\eta \,k_{T\,P\,R}\,p_{T\,P\,R}\,M\,A\,P_{t\,a\,r\,g\,e\,t}\right]$$

The problem of individualizing the physiological model can be formulated as solving the following maximum *a posterioi* estimation problem (Gelman et al., 2013):

$${\overset{˘}{\theta }}_{i}=\text{arg}\,\max_{\theta }P\,\left(\theta |U_{i}^{d},Y_{i}^{d}\right)=\text{arg}\,\max_{\theta }P\,\left(Y_{i}^{d}|U_{i}^{d},\theta \right)\,P\,\left(\theta \right)$$

$$=\text{arg}\,\min_{\theta }\left[-\log P\,\left(Y_{i}^{d}|U_{i}^{d},\theta \right)-\log P\,\left(\theta \right)\right]$$

(8) $$=\text{arg}\,\min_{\theta }\left[J_{1}\,\left(\theta \right)+J_{2}\,\left(\theta \right)\right]$$

where ${\overset{˘}{\theta }}_{i}$ is the vector of model parameters associated with the individual *i*, $J_{1}\,\left(\theta \right)=-\log P\,\left(Y_{i}^{d}|U_{i}^{d},\theta \right)$ corresponds to the likelihood of the individual-specific data with respect to the model output, and *J*_2_(θ) = −*log*⁡*P*(θ) is a regularization term that corresponds to prior knowledge *P*(θ) about the parameter values. Assuming that $P\,\left(Y_{i}^{d}|U_{i}^{d},\theta \right)$ is a Gaussian distribution due to noise on the output measurements, we can set *J*_1_(θ) as the covariance-weighted mean-squared output errors:

(9) $$J_{1}\,\left(\theta \right)=\left[Y_{i}\,\left(U_{i}^{d},\theta \right)-Y_{i}^{d}\right]\,{\text{Σ}}^{-1}\,{\left[Y_{i}\,\left(U_{i}^{d},\theta \right)-Y_{i}^{d}\right]}^{T}$$

In case there is no informative prior knowledge about the probability distribution of the model parameters, *J*_2_(θ) in Eq. 8 does not depend on θ and is effectively removed. However, in that case, the optimization problem in Eq. 8 is likely to become ill-posed and suffer from practical identifiability challenges when the physiological model is too complex relative to the available individual-specific data (which is frequently the case).

The main idea to address the practical identifiability problem in the absence of sufficient individual-specific data is to construct an appropriate prior distribution *P*(θ) to additionally inform the modeling problem. This prior distribution can be constructed based on the prior knowledge about plausible parameter ranges as well as heterogeneous data from a population of individuals. In this work, we formulate a prior in the shape of a Laplace distribution of the following form:

(10) $$P\,\left(\theta \right)\propto \prod_{m=1}^{n_{\theta }}\exp\left(-\frac{\left|\theta_{m}-{\bar{\theta }}_{m}\right|}{b_{m}}\right)\to J_{2}\,\left(\theta \right)=\lambda \,\sum_{m=1}^{n_{\theta }}\frac{1}{b_{m}}\,\left|\theta_{m}-{\bar{\theta }}_{m}\right|$$

where θ_*m*_ is the *m*-th element in θ, ${\bar{\theta }}_{m}$ is the *m*-th element of the mode of the distribution $\bar{\theta }$, λ is the regularization weight, and *b*_*m*_ is a normalization factor for θ_*m*_, which reflects prior knowledge about reasonable and/or physiological ranges for the model parameter [i.e., given physiological upper $\left({\hat{\theta }}_{m}\right)$ and lower $\left({\overset{ˇ}{\theta }}_{m}\right)$ bounds on θ_*m*_, we set $b_{m}=\left({\hat{\theta }}_{m}-{\overset{ˇ}{\theta }}_{m}\right)$; see Appendix Table A1 for ${\hat{\theta }}_{m}$ and ${\overset{ˇ}{\theta }}_{m}$ associated with the physiological model in section “Cardiovascular Hemodynamics in Hemorrhage and Circulatory Resuscitation”]. Equation 10 is a form of *L*_*1*_-regularization (Bach et al., 2011; Jenatton et al., 2011), which represents the inter-individual variability observed in the population data by compressed parametric deviations from a mode denoted by $\bar{\theta }$. This representation may be possible if the studied physiological modeling problem has the sloppiness property (Machta et al., 2013; Transtrum et al., 2015), i.e., a wide range of parameter sensitivities, implying compressibility (see section “Role of Regularization in Individualizing Physiological Models” for more details as far as our case study is concerned).

Having Eq. 10, the problem of constructing the prior reduces to determining the parameters λ and $\bar{\theta }$ from heterogeneous population data. Techniques exist in the field of non-linear mixed-effects modeling (Davidian and Giltinan, 2003) and system identification (Pan et al., 2018) that can deal with such problems, often with comparable results (Kataria et al., 1994; Hahn et al., 2011). In this work, we define $\bar{\theta }$ as the solution to the following maximum-likelihood optimization problem based on the population data from *L* = *N*−1 individuals:

(11) $$\bar{\theta }=\text{arg}\,\min_{\theta }\sum_{i=1}^{L}\left[Y_{i}\,\left(U_{i}^{d},\theta \right)-Y_{i}^{d}\right]\,{\text{Σ}}^{-1}\,{\left[Y_{i}\,\left(U_{i}^{d},\theta \right)-Y_{i}^{d}\right]}^{T}$$

Note that the population-based modeling problem in Eq. 11 is supposedly less susceptible to practical identifiability challenges than the individualization problem in Eq. 8 by virtue of the larger amount of heterogeneous data available from the population. The resulting model with $\bar{\theta }$ will hereafter be called the population-average model, which is intended to represent an aggregated and sensible model of typical physiological behavior.

**FIGURE 2:**
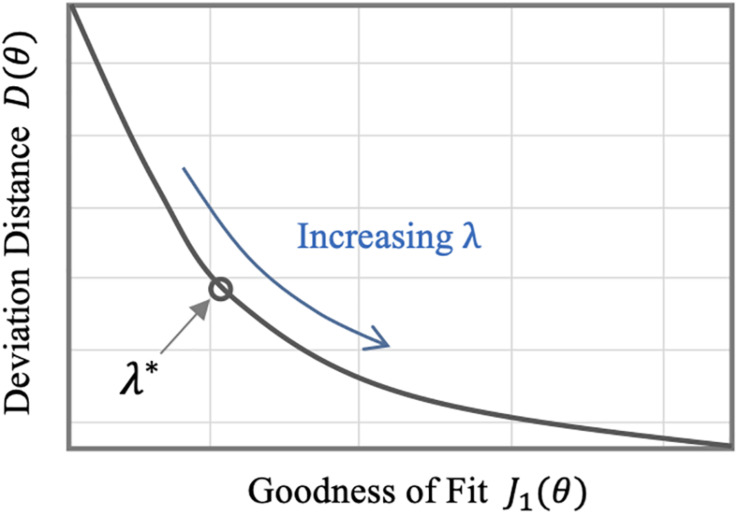
Typical trend of the goodness of fit metric *J*_*1*_ and the deviation distance *D* with respect to λ in individualizing physiological models.

The regularization weight λ serves as the relative scaling factor between the spread of the prior in Eq. 10 and the spread of the likelihood in Eq. 9. To find an appropriate individual-specific weight $\lambda_{i}^{*}$, we adopt an approach related to the L-curve method (Hansen, 1992; Hansen and O’Leary, 1993). The weight $\lambda_{i}^{*}$ may be estimated by solving the optimization problem in Eq. 8 for a range of *λ* ∈ [0*λ*_*max*_] and plotting the resulting values for the goodness of fit cost *J*_1_(θ) and the deviation distance *D*(θ) = *J*_2_(θ)/λ (Figure 2). An initial increase of λ from zero tends to decrease *D* by compressing parametric deviations from $\bar{\theta }$ in low-sensitivity directions. This increases *J*_*1*_, but the extent is not large relative to the decrease in *D* since the effect of restricting low-sensitivity (i.e., sloppy) deviations on *J*_*1*_ is, by definition, small. This trend continues until λ reaches a critical value. However, beyond this critical value, sensitive deviations also become restricted, leading to a substantial increase in *J*_*1*_ relative to the decrease in *D*. The appropriate $\lambda_{i}^{*}$ can be chosen as the value of λ in between these regime changes (Figure 2).

**FIGURE 3:**
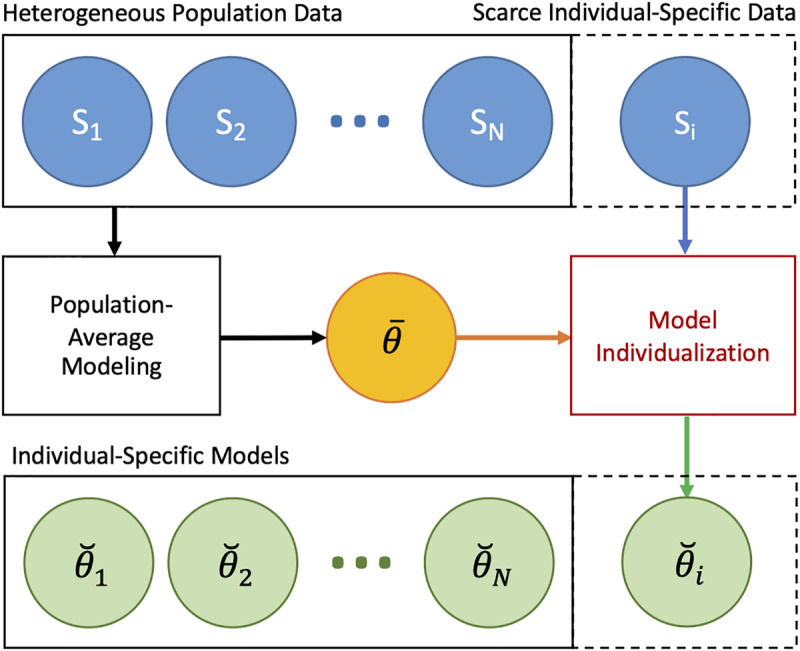
Individualizing physiological models with scarce data *via* regularization. In the first step, a prior (regularization) is constructed from heterogeneous data measured in a population by solving Eq. 11. In the second step, the physiological model is individualized with regularization using scarce data by solving Eq. 8.

In sum, individualizing a physiological model *via* regularization can be performed in two steps: (i) constructing a prior (regularization function) from heterogeneous data measured in a population by solving Eq. 11 and (ii) individualizing the physiological model with regularization using scarce individual-specific data by solving Eq. 8 (Figure 3).

### Data Analysis

We applied the individualization procedure outlined in section “Individualizing the Physiological Model” to the physiological model outlined in section “Cardiovascular Hemodynamics in Hemorrhage and Circulatory Resuscitation”. In particular, we constructed an appropriate regularization function and individualized the physiological model under varying levels of scarcity in the individual-specific data. To enable the data analysis with relatively small sample size, we performed a leave-one-out type of data analysis (details follow).

We individualized the physiological model under three levels of increasing data scarcity: (i) case 1, in which the physiological model was individualized given BV, CO, and MAP data, (ii) case 2, in which the physiological model was individualized given CO and MAP data, and (iii) case 3, in which the model was individualized given only MAP data. We considered these data scarcity scenarios for two investigational reasons: (i) comparison of the physiological models individualized with and without the use of regularization in the three cases may offer insight into how regularization affects the parameter values in response to varying data scarcity and (ii) analysis of the prediction errors for unmeasured internal states (i.e., BV in case 2 and BV and CO in case 3) with and without the use of regularization may offer insight into how regularization affects the physiological plausibility and the relevance of the physiological model under data scarcity.

For each animal subject, we analyzed the data pertaining to the rest of the *L* = 22 animal subjects with the maximum-likelihood method in Eq. 11 to derive a population-average physiological model, characterized by $\bar{\theta }$, that predicts BV, CO, and MAP responses to hemorrhage and fluid resuscitation inputs. Then, we individualized the physiological model to the (excluded) animal *via* regularization by solving Eq. 8 with Eq. 10 as the regularization function. We selected the appropriate value of λ for each individual subject as described in section “Individualizing the Physiological Model” (Figure 2). We repeated individualizing the physiological model in this way for the three dataset scarcity cases. For comparative analysis, we also individualized the physiological model to each animal without using regularization for all the three data scarcity scenarios.

With the goal of assessing the plausibility and the relevance of the physiological models, we compared the models individualized with and without regularization as well as the population-average model. We devised two quantitative error metrics for this purpose: (i) output prediction error *e*_*1*_, defined as the normalized root-mean-square error (RMSE) between model-predicted and measured output signals (among BV, CO, and MAP) explicitly used for individualizing the physiological model and (ii) state prediction error *e*_*2*_, defined as the normalized RMSE between model-predicted and measured internal state signals not used in individualizing the physiological model. For example, in case 3, *e*_*1*_ is the normalized RMSE associated with MAP:

(12) $$e_{1}=\frac{1}{N\,n_{M\,A\,P}^{1/2}}\,\sum_{i=1}^{N}$$

$$\left[\frac{1}{\sigma_{M\,A\,P}}\,{∥M\,A\,P_{i}\,\left(t_{1},\ldots ,t_{n_{M\,A\,P}}\right)-M\,A\,P_{i}^{d}\,\left(t_{1},\ldots ,t_{n_{M\,A\,P}}\right)∥}_{2}\right]$$

while *e*_*2*_ is the normalized RMSE associated with BV and CO:

(13) $$e_{2}=\frac{1}{N\,{\left(n_{B\,V}+n_{C\,O}\right)}^{1/2}}\sum_{i=1}^{N}[(\frac{1}{\sigma_{B\,V}^{2}}∥BV_{i}\left(t_{1},\ldots ,t_{n_{B\,V}}\right).$$

$$-{B\,V_{i}^{d}\,\left(t_{1},\ldots ,t_{n_{B\,V}}\right)∥}_{2}^{2}+\frac{1}{\sigma_{C\,O}^{2}}∥C\,O_{i}\,\left(t_{1},\ldots ,t_{n_{C\,O}}\right).$$

$${-CO_{i}^{d}\left(t_{1},\ldots ,t_{n_{C\,O}}\right){∥}_{2}^{2})}^{1/2}]$$

We employed the Wilcoxon signed-rank test in conjunction with the Bonferroni correction to assess the statistical significance of the difference in these metrics between the models.

A numerical simulation of the physiological model was performed using MATLAB^®^’s ODE solvers in the Simulink^®^ environment. The numerical optimization was performed using MATLAB^®^’s Optimization Toolbox. Data analysis and visualization was performed using the seaborn and the matplotlib libraries in Python.

## Results

**FIGURE 4:**
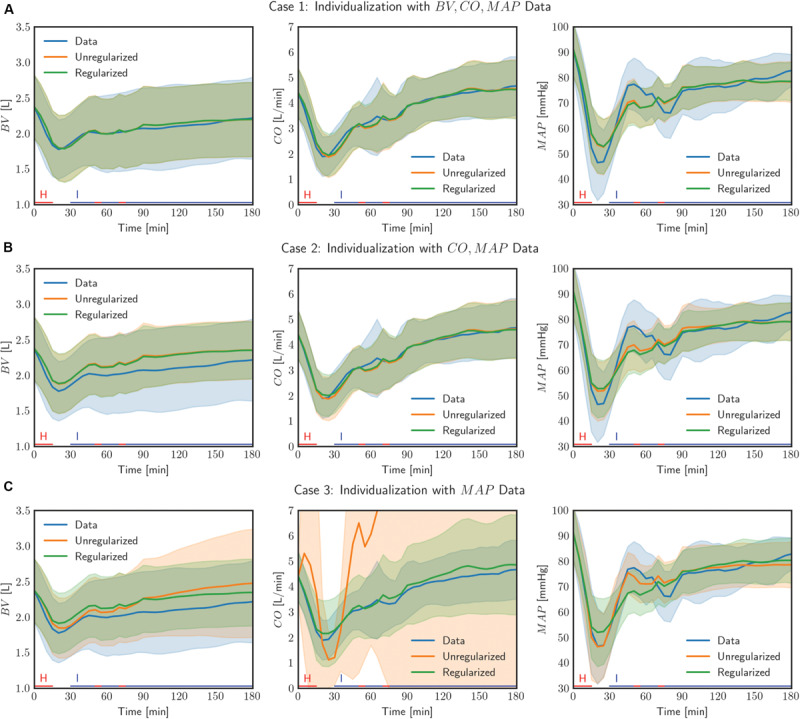
Comparison of measured data with model outputs in all (*N* = 23) individualized models, with and without the use of regularization, in three cases of data scarcity. Solid lines correspond to the mean response across all individuals and the shaded bands show the standard deviation of the response across all individuals. The lines on the horizontal axis indicate the timings of hemorrhage and infusion in the experimental protocol.

**FIGURE 5:**
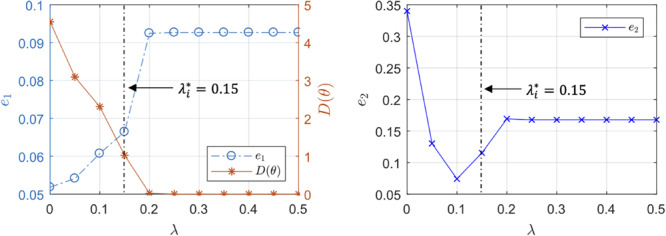
Representative trends of the output prediction error *e*_*1*_, the internal state prediction error *e*_*2*_, and the parametric deviation distance in a subject with respect to the regularization weight λ.

**FIGURE 6:**
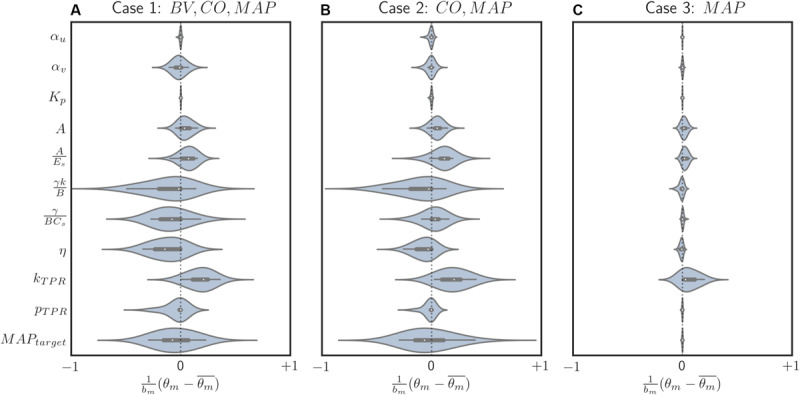
Box and violin (density estimate) plots of regularized parametric deviations from the population-average values across all individualized models, with respect to varying levels of data scarcity. **(A)** Case 1 (individualization with BV, CO, and MAP data). **(B)** Case 2 (individualization with CO and MAP data). **(C)** Case 3 (individualization with MAP data). Horizontal axis: normalized parametric deviations from the population-average values, where model parameters were normalized according to their nominal range (*b*_*m*_’s in Eq. 10; see Appendix Table A1). Vertical axis: model parameters.

Table 2 shows the output (*e*_*1*_) and the internal state (*e*_*2*_) prediction errors associated with the population-average model and the individualized models (both with and without the use of regularization), while Figure 4 presents the measured *versus* the model-predicted output and internal state signals associated with the population-average and the individualized physiological models. Figure 5 presents the representative behaviors of the output prediction error (*e*_*1*_), the internal state prediction error (*e*_*2*_), and the parametric deviation distance (*D*) with respect to the regularization weight λ in an individual subject. Figure 6 presents the regularized parametric deviations from the population-average model with respect to varying degrees of data scarcity.

**TABLE 2:** Output and state prediction errors associated with population-average model as well as individualized models with and without the use of regularization [mean (SD), *N* = 23].

	**Population average**	**Individualized/no regularization (λ = 0)**	**Individualized/regularization**
**Case 1: individualizing the physiological model with BV, CO, and MAP (individualized/regularization, λ = 0.10(0.06))**			
BV (L)	0.26 (0.19)	0.10 (0.03)	0.10 (0.04)
CO (L/m)	1.31 (0.80)	0.34 (0.18)	0.34 (0.17)
MAP (mmHg)	17.1 (8.88)	7.23 (2.01)	7.40 (2.05)
*e*_*1*_	0.25 (0.14)	0.08 (0.02)^†^	0.08 (0.02)^†^
**Case 2: individualizing the physiological model with CO and MAP (individualized/regularization, λ = 0.16(0.13))**			
BV (L)	0.26 (0.19)	0.29 (0.21)	0.25 (0.18)
CO (L/m)	1.31 (0.80)	0.30 (0.18)	0.32 (0.15)
MAP [mmHg]	17.1 (8.88)	6.94 (2.20)	7.92 (2.42)
*e*_*1*_	0.30 (0.19)	0.09 (0.03)^†^	0.10 (0.03)^†^
*e*_*2*_	0.15 (0.15)	0.16 (0.17)	0.14 (0.14)
**Case 3: individualizing the physiological model with MAP (individualized/regularization, λ = 0.22(0.17))**			
BV (L)	0.26 (0.19)	0.43 (0.35)	0.27 (0.18)
CO (L/m)	1.31 (0.80)	13.0 (18.0)	1.19 (0.94)
MAP (mmHg)	17.1 (8.88)	4.85 (1.43)	7.48 (3.03)
*e*_*1*_	0.24 (0.13)	0.07 (0.02)^†^	0.10 (0.04)^†^
*e*_*2*_	0.26 (0.15)*	2.08 (2.83)	0.24 (0.20)*

*p < 0.016 compared to the individualized model without the use of regularization. ^†^*p* < 0.016 compared to the population-average model.

## Discussion

With the long-term goal of advancing the future development and the testing of clinical care automation based on physiological models, we sought to investigate the role of regularization in individualizing a physiological model using scarce data. Our findings from the case study of blood volume kinetics and cardiovascular hemodynamics in hemorrhage and circulatory resuscitation suggest that regularization can be effective in individualizing the physiological model to capture salient individual-specific characteristics and restrict unnecessary deviations from the population-average model. Furthermore, regularization results in appropriately varying parametric deviations to cope with the varying levels of data scarcity, thereby securing the physiological plausibility and the relevance of the individualized physiological model (details follow).

### Role of Regularization in Individualizing Physiological Models

First, the use of regularization effectively maintained the goodness of fit to individual-specific data while reducing parametric deviations in insensitive directions. In all scenarios associated with the three levels of data scarcity (see section “Data Analysis”), the difference in *e*_*1*_ was small between the physiological models individualized with and without the use of regularization (Table 2 and Figure 4). In contrast, the parametric deviations [in terms of the distance *D*(θ) = *J*_2_(θ)/λ averaged across all the animals] were smaller when regularization was employed (1.19 in case 1, 1.14 in case 2, and 0.30 in case 3) than when it was not (1.87 in case 1, 1.89 in case 2, and 2.82 in case 3). This suggests that regularization assists in individualizing a physiological model to achieve an adequate level of goodness of fit while restricting parametric deviations that may only result in overfitting.

Second, the physiological model individualized with the use of regularization exhibited improved physiological plausibility and relevance in comparison with the one individualized without the use of regularization in terms of the accuracy in predicting internal state signals. In general, the goodness of fit associated with BV and CO responses was deteriorated when the corresponding measurement was removed in individualizing the physiological model. Figure 4 shows that indeed (i) BV prediction is deteriorated when BV data become unavailable (case 1 → case 2 and case 3) and (ii) CO prediction is likewise deteriorated when CO data become unavailable (case 2 → case 3). In case 2, *e*_*2*_ (i.e., BV prediction error) was smaller when regularization was employed than when it was not employed (*p* = 0.03) (Table 2). In case 3, *e*_*2*_ (i.e., BV and CO prediction errors) was likewise smaller when regularization was employed than when it was not employed (*p* < 0.01) (Table 2). Interestingly, MAP prediction was notably improved as data became more scarce when regularization was not employed [case 1 (7.23 mmHg) to case 2 (6.94 mmHg) to case 3 (4.85 mmHg), all in terms of mean errors; see Table 2]. Thus, the large deterioration in *e*_*2*_ may be attributed to overfitting to MAP data. In conjunction with the finding above on the role of regularization in restricting unnecessary parametric deviations, this suggests that regularization improves the ability of the individualized physiological model to predict the internal states accurately by preventing the undesired over-tuning of model parameters away from their population-average values. One additional minor note is that BV prediction achieved with regularization was comparable between case 2 and case 3. This can be interpreted as follows: (i) both case 2 and case 3 did not use BV data in individualizing the physiological model, and regularization tended to reduce the BV component in the physiological model to the population-average model (as can be seen by the comparable BV prediction errors associated with these models; see Table 2) and (ii) presumably CO data did not provide much implications on the behavior of BV on top of the prior knowledge [as can be seen by the BV prediction errors in case 2 with and without the use of regularization (0.25 L and 0.29 L, both in terms of mean errors; see Table 2].

Third, the individualized physiological model exhibited goodness of fit parametric deviation behavior with respect to the regularization weight λ as anticipated in Figure 2, especially in the case of very scarce data (case 3; see Figure 5). A sharp initial decrease in *D*(θ) = *J*_2_(θ)/λ relative to a modest increase in *e*_*1*_ was observed when λ was initially increased from zero. Further increase in λ after a critical point caused *e*_*1*_ to largely increase relative to a steady decrease in the parametric deviation distance and eventually approach that of the population-average model. In sum, an appropriate $\lambda_{i}^{*}$ could be selected as outlined in section “Individualizing the Physiological Model” (λ^∗^ = 0.15). Interestingly, there appeared to be a notable link between the behaviors of *D*(θ) and *e*_*2*_: *e*_*2*_ initially showed a notably decreasing trend similar to that of *D*(θ), which suggests that regularization improves the ability of the individualized model to predict internal states (Figure 5). For larger values of λ, *e*_*2*_ increased, which can be attributed to the restriction of sensitive parametric deviations, degenerating the individualized model to the population-average model.

Fourth, the physiological model individualized with the use of regularization was associated with a significantly superior output prediction error and a comparable internal state prediction error in comparison with the population-average model (Table 2). This suggests that regularization is effective in judiciously individualizing sensitive model parameters to achieve desirable goodness of fit to individual-specific data while at the same time capitalizing on prior knowledge (in the form of regularization) to preserve the ability to adequately predict unmeasured internal states.

### Parametric Deviations in Relation to Varying Data Scarcity

In our case study, regularization-induced deviations from the population-average model showed two noteworthy trends. First, with regularization, deviations tended to decrease as the data became more scarce (Figure 6). In other words, (i) regularization allowed meaningful deviations if ample data were available so that the physiological model could be better individualized by absorbing the individual-specific characteristics represented by the data, whereas (ii) it restricted unwarranted deviations if scarce data were available so that the physiological model could fall back to the population-average model. This can be viewed as a desirable behavior in that increasingly leveraging the prior knowledge (i.e., the population-average model) as the data scarcity increases is the intended effect of regularization. In contrast, such a parametric deviation trend was not observed when regularization was not used. The opposite occurred instead: scarce data resulted in more aggressive deviations from the population-average model (see the deviation distance results reported in section “Role of Regularization in Individualizing Physiological Models”), which consequently compromised the fidelity of the individualized physiological models (e.g., its internal state prediction was deteriorated; see Table 2). Second, regularization appeared to effectively individualize those parameters relevant to the presented data. For example, the most visibly deviated parameter in case 3 was the one associated with BP regulation (*k*_*TPR*_), which is reasonable in that only MAP measurements were presented to individualize the physiological model in case 3 (Figure 6C). The parameters associated with both CV function (γ/*BC*_*s*_ and η) and BP regulation (*k*_*TPR*_ and *MAP*_*target*_) were likewise visibly deviated in case 2, in which CO and MAP measurements were presented (Figure 6B). Finally, the parameters associated with BV were likewise largely deviated (α_*v*_, in particular) when BV measurements were presented in addition to CO and MAP data (Figure 6A). One caveat is that, despite the presence of these trends, the studied parameters belong to a complex physiological system and it may not be possible to attribute each parametric deviation completely to the availability of a specific type of measurement.

### Study Limitations

This work has limitations. First, this work investigated the role of regularization in a specific case study. Hence, the insights obtained from the case study may not be universally true in all physiological modeling problems. In particular, the legitimacy of the key assumption used to address the adverse effect of scarce data on the quality of individualized models (that the inter-individual variability observed in the population data can be represented by compressed deviations from a population-average model according to the Laplace distribution in Eq. 10) may depend on the specificity of the physiological modeling problem at hand. On the one hand, such a compressed representation may indeed be valid in many real-world physiological modeling problems with the sloppiness property (Machta et al., 2013; Transtrum et al., 2015) (which implies parameter-space compressibility). On the other hand, regularization may not prove effective if the quality of the data and the model structure are such that the majority of the model parameters are associated with sensitive deviations (where individualizing the physiological model requires deviations in all the model parameters; in this case, regularization is not needed) or insensitive deviations (where a population-average model may suffice because of negligible inter-individual variability). In any case, the validity of the “parameter-space compressibility” assumption in a specific physiological modeling application can be assessed based on model prediction errors and parameter values in the individualized physiological models: the bias introduced by the inadequacy of the assumption will manifest itself as poor goodness of fit of the individualized physiological model to the data, while a lack of compressibility will manifest itself as estimated elements of ${\overset{˘}{\theta }}_{i}$ in Eq. 8 that all deviate from $\bar{\theta }$ in Eq. 11. Second, regularization does not guarantee, and may even prevent, the convergence of the physiological model to the “true” individual-specific physiological model. Indeed its main purpose is to minimize the deviations from the prior knowledge (i.e., population-average model). Thus, the quality of a physiological model individualized with regularization, especially using highly scarce data, hinges upon the quality of prior knowledge. Hence, the physiological model individualized with regularization is a candidate approximation to the ideal individual-specific physiological model (which would be obtained with sufficiently diverse and high-quality data obtained from an individual). Taking these aspects into account, regularization may be viewed as an effective tool to individualize a physiological model in cases with a relatively complex model and scarce individual-specific data.

## Conclusion

We sought to garner potentially generalizable insight into the role of regularization as prior knowledge in individualizing physiological models using scarce data. The findings obtained from a clinically significant case study illustrated that regularized individualization creates physiological models equipped with several desirable properties: (i) an adequate trade-off between goodness of fit to scarce individual-specific data and deviation from a population-average model, (ii) improved physiological plausibility and relevance, and (iii) parametric deviations relevant to the scarcity level of the data. Noting that regularization is not prevalently used in the physiological modeling domain, future work must be devoted to exploring the use of regularization in individualizing a range of physiological models and developing appropriate theory and novel structures for the regularization functions based on appropriate physiological assumptions.

## Data Availability Statement

The datasets analyzed in this study can be obtained from Dr. George C. Kramer at the University of Texas Medical Branch (gkramer@utmb.edu).

## Author Contributions

AT, CS, and J-OH formulated the procedure to individualize the physiological model *via* regularization and wrote and revised the manuscript. XJ, AL, CS, and J-OH developed the physiological model. AT and J-OH analyzed the data. XJ and AL reviewed the manuscript.

## Conflict of Interest

The authors declare that the research was conducted in the absence of any commercial or financial relationships that could be construed as a potential conflict of interest.

## Appendix

**TABLE 3:** List of lumped parameters individualized in the physiological model with nominal upper bounds, lower bounds, and population-average values.

**Model parameter**	**Unit**	**Lower bound (${\overset{ˇ}{\theta }}_{m}$)**	**Upper bound (${\overset{ˇ}{\theta }}_{m}$)**	**Population-average value ($\bar{\theta }$)**
α_*u*_	(1)	0	6	4.29
α_*v*_	(1)	0	6	2.28
*K*_*p*_	(1/min)	10^–2^	0.5	0.21
*A*	(1/L)	0	0.3	0.23
*A*/*E*_*s*_	(1/mmHg)	0	10^–3^	2.54 × 10^–4^
γ*k*/*B*	(1/mmHg)	0	10^–4^	3.62 × 10^–5^
γ/*B**C*_*s*_	(1/L)	0	0.1	2.43 × 10^–2^
η	(1)	0.2	1	0.43
*k*_*TPR*_	(1/L)	0	1	5.87 × 10^–2^
*p*_*TPR*_	(1/min)	0	1	0.66
*MAP*_*target*_	(mmHg)	40	100	70.1
